# Melorheostosis with Extraosseous Extension Mimicking a Cartilaginous Tumor: A Case Report

**DOI:** 10.5334/jbsr.4139

**Published:** 2025-11-10

**Authors:** Olympia Lemontzis

**Affiliations:** 1 ULB, Lenniksebaan 808, 1070 Brussels, Belgium

**Keywords:** knee pain, melorheostosis, dripping candle wax, extra-osseous extension, radioclinical correlation, incidentaloma

## Abstract

*Teaching point:* Melorheostosis may exceptionally present with extraosseous extension mimicking a cartilaginous tumor, emphasizing the crucial role of anatomo-radiological correlation when histology is confusing.

## Case History

A 65-year-old patient presented with chronic and disabling knee pain associated with progressive joint stiffness. The pain was mainly mechanical, with posterior discomfort limiting flexion to 60°. Clinical examination revealed a hard-consistency mass located in the popliteal fossa as well as a limb length discrepancy. The patient’s medical history included hypercholesterolemia, hyperuricemia, and treated type 2 diabetes.

**Figure 1:**
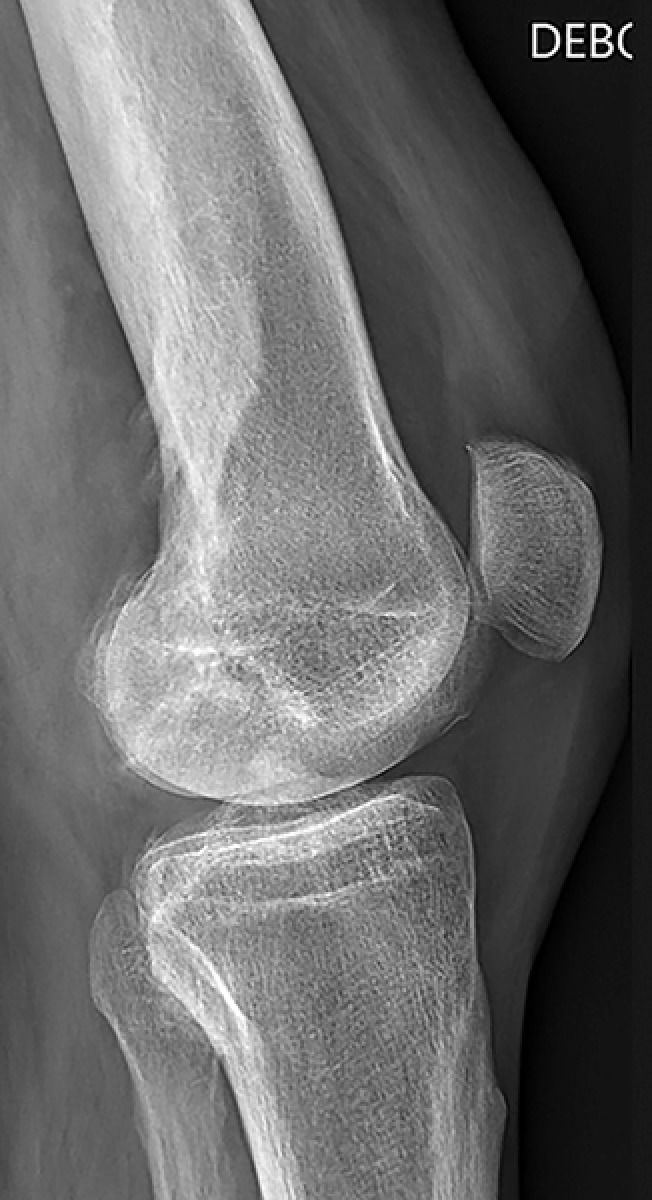
Knee X-Ray: femoral cortical thickening “dripping candle wax”.

**Figure 2:**
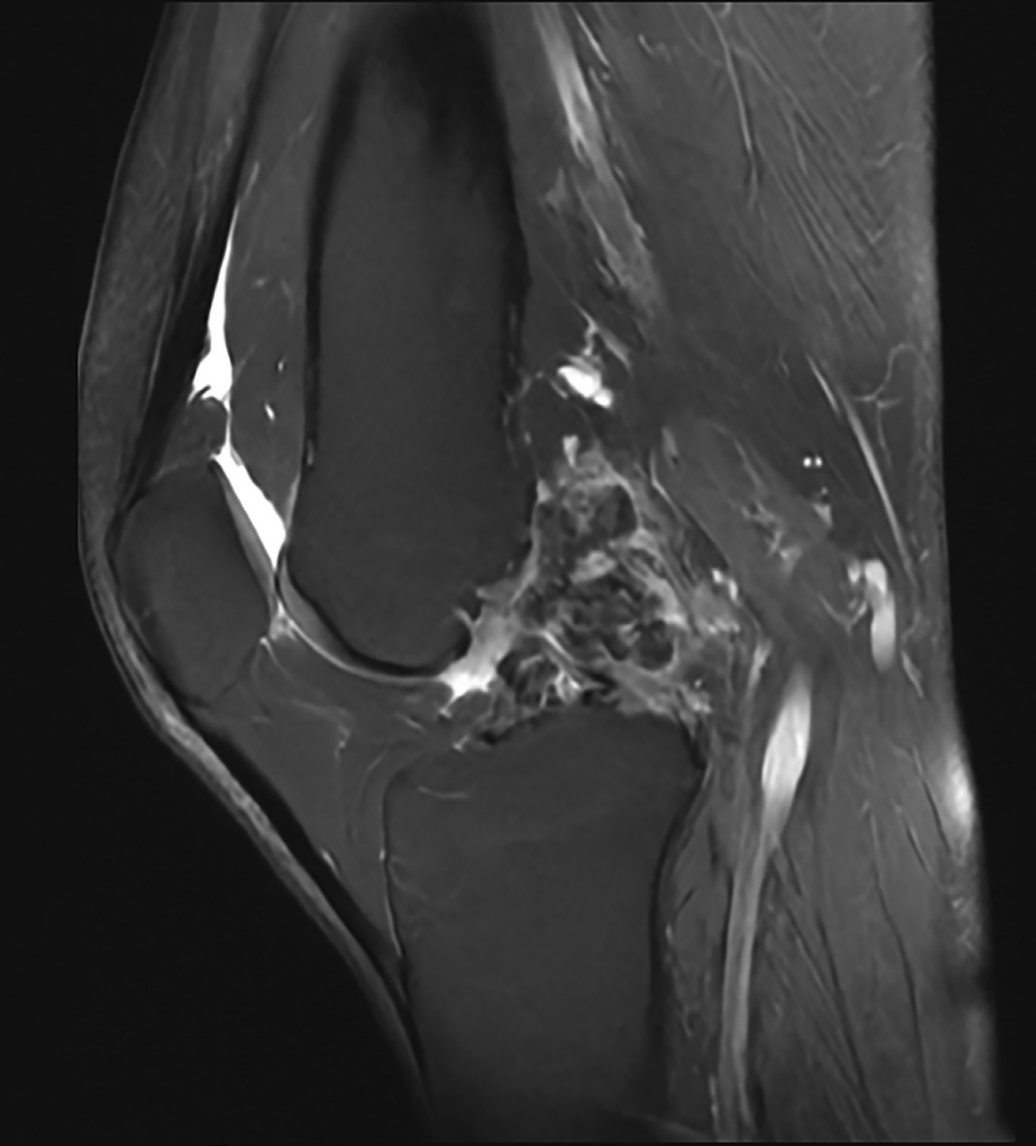
MRI: hypertrophy cortex with low DP fat-saturated signal.

**Figure 3:**
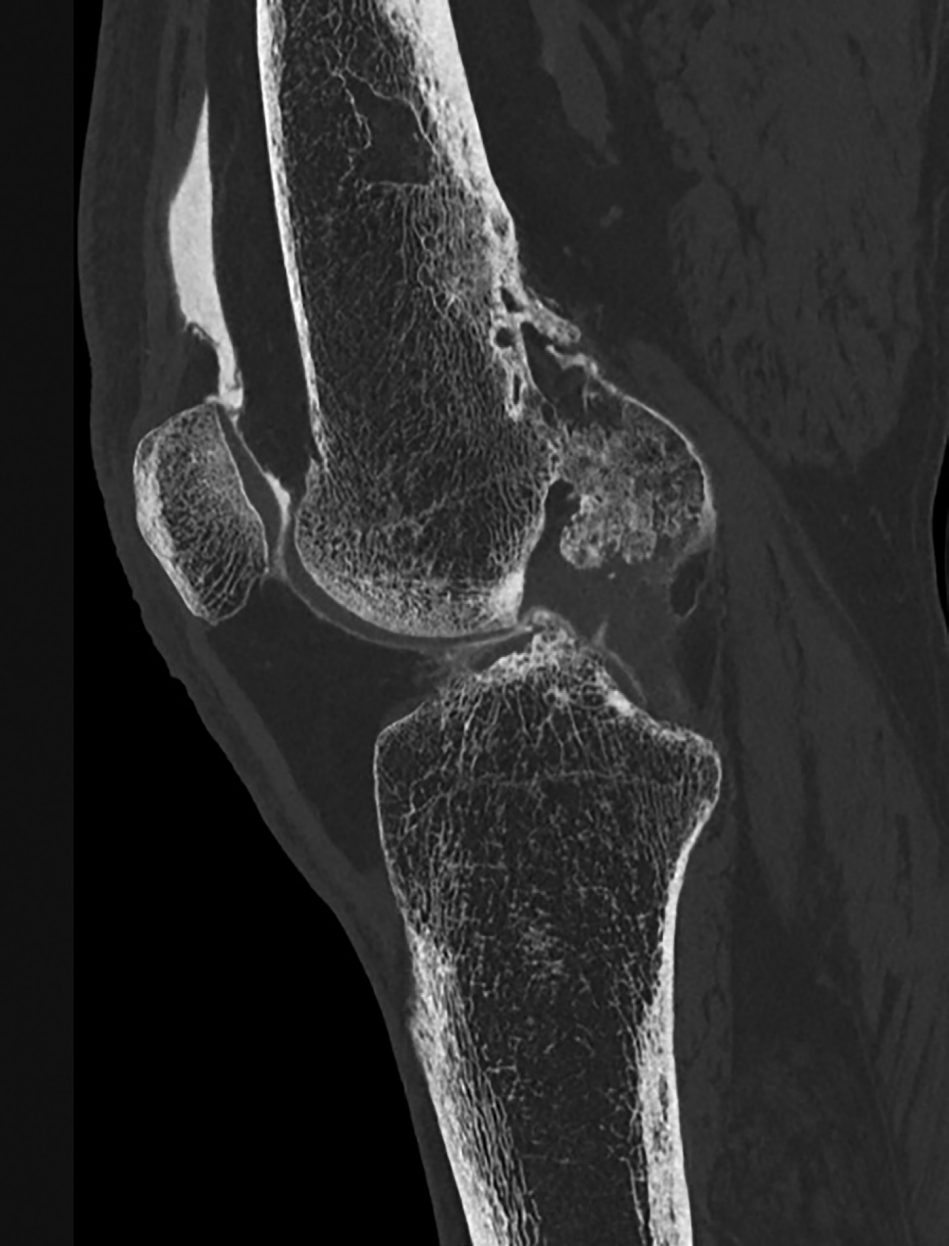
Arthro-CT: osseous extension into posterior intercondylar notch.

Lateral radiograph showed cortical thickening of the femur with a “dripping candle wax” appearance, typical of melorheostosis (Figure 1). Given the clearly osseous nature of the lesion, an ultrasound was not deemed necessary. Magnetic Resonance imaging revealed a hypertrophic cortex with low signal intensity on T1 and DP fatsat sequences (Figure 2), corresponding to sclerotic bone extending into the adjacent soft tissues, associated with peripheral enhancement after gadolinium injection. Computed tomography arthrography further detailed these abnormalities, showing an ossification partially incorporated into the posterior aspect of the intercondylar notch, corresponding to the extraosseous extension of the melorheostotic lesion (Figure 3).

After imaging established the benign nature of the lesion, with no signs of local aggressiveness or cortical destruction, a CT-guided biopsy was subsequently performed. Its purpose was to rule out a giant cell tumor or synovial chondromatosis, initially considered among the differential diagnoses. Histology revealed an osteocartilaginous lesion compatible with primary synovial osteochondromatosis, suggesting a cartilaginous tumor. However, radiological findings allowed the diagnosis of melorheostosis with extraosseous extension to be re-established. Long-term clinical and radiological follow-up showed no progression or new lesion, confirming the benign nature of the condition.

## Comment

Melorheostosis is a hyperostosis characterized by irregular cortical thickening, giving the typical radiographic appearance of “dripping candle wax.” Clinical presentation is variable, ranging from incidental discovery to chronic pain or deformity. A particular feature, illustrated in our case, is the extension of the lesion into periarticular soft tissues. This can lead to diagnostic confusion with other conditions, notably synovial chondromatosis.

Diagnosis is primarily based on standard radiography, which demonstrates the typical cortical hyperostosis sometimes associated with ossified soft-tissue masses. Histology, however, may be misleading due to a reactive osteocartilaginous component. In this context, radioclinical correlation remains essential to support the diagnosis.

This case thus illustrates the importance of a multidisciplinary approach and anatomo-radiological comparison in the evaluation of bone lesions. Imaging retains a major discriminative value through its characteristic morphological features, even when histology suggests a tumoral lesion.

Genetic abnormalities, including LEMD3 and MAP2K1 mutations, have been reported and may aid in confirming the diagnosis in uncertain cases. Therefore, in atypical presentations or when discrepancies exist between imaging and histology, targeted genetic testing may be considered to confirm the diagnosis.

Treatment remains essentially symptomatic, based on pain management, physiotherapy, and, in certain cases, surgical intervention [1].
